# Implementing ERAS: how we achieved success within an anesthesia department

**DOI:** 10.1186/s12871-021-01260-6

**Published:** 2021-02-05

**Authors:** Dan B. Ellis, Aalok Agarwala, Elena Cavallo, Pam Linov, Michael K. Hidrue, Marcela G. del Carmen, Rachel Sisodia

**Affiliations:** 1 Department of Anesthesia, Critical Care, and Pain Medicine, Massachusetts General Hospital, 55 Fruit Street, Boston, MA 02114 USA; 2 Department of Anesthesia, Massachusetts Eye and Ear Infirmary, Boston, USA; 3 Department of Anesthesia, Critical Care, and Pain Medicine, Massachusetts General Hospital, 55 Fruit Street, Boston, MA 02114 USA; 4 Massachusetts General Physicians Organization, Massachusetts General Hospital, 55 Fruit Street, Boston, Massachusetts 02114 USA; 5 Department of Gynecology Oncology, Massachusetts General Hospital, 55 Fruit Street, Boston, MA 02114 USA

**Keywords:** Enhanced recovery after surgery, Gabapentin, PACU

## Abstract

**Background:**

The Massachusetts General Hospital is a large, quaternary care institution with 58 operating rooms, 164 anesthesiologists, 76 certified nurse anesthetists (CRNAs), an anesthesiology residency program that admits 25 residents annually, and 35 surgeons who perform laparoscopic, vaginal, and open hysterectomies. In March of 2018, our institution launched an Enhanced Recovery After Surgery (ERAS) pathway for patients undergoing hysterectomy. To implement the anesthesia bundle of this pathway, an intensive 14-month educational endeavor was created and put into effect. There were no subsequent additional educational interventions.

**Methods:**

We retrospectively reviewed records of 2570 patients who underwent hysterectomy between October 2016 and March 2020 to determine adherence to the anesthesia bundle of the ERAS Hysterectomy pathway. RESULTS: Increased adherence to the four elements of the anesthesia bundle (*p* < 0.001) was achieved during the intervention period. Compliance with the pathway was sustained in the post-intervention period despite no additional actions.

**Conclusions:**

Implementing the anesthesia bundle of an ERAS pathway in a large anesthesia group with diverse providers successfully occurred using implementation science-based approach of intense interventions, and these results were maintained after the intervention ceased.

## Background

Enhanced Recovery After Surgery (ERAS) is one of the most significant innovations in perioperative care.[1] As hospitals streamline care and move increasingly more complex surgeries to outpatient surgical centers, efficiently and safely moving patients through the perioperative environment is of utmost importance.[2–6] Increased adherence to ERAS pathways has repeatedly shown decreased complications and reduced overall perioperative costs.[5, 7–10]

Implementing and maintaining strict adherence to ERAS pathways is challenging, and compliance values of approximately 70% are common.[11] Additionally, as greater numbers of providers become involved in ERAS care, achieving high compliance rates with ERAS pathways becomes more complex. This challenge is pronounced in large anesthesia practices, and in academic centers with varying patient acuities and staff of different levels. The national trend of large anesthesia groups covering multiple anesthetizing locations with diverse provider groups also adds to this complexity. To date, the literature describes multidisciplinary approaches to implementing ERAS bundles. However, a step-by-step approach to implementing and maintaining compliance with anesthesia bundles in large anesthesia practices has not been thoroughly described.[12–15]

As part of a quality improvement project, in March of 2018, an Enhanced Recovery After Surgery pathway for laparoscopic, vaginal, and open hysterectomy patients called “ERAS Hysterectomy” was implemented at the Massachusetts General Hospital. This pathway was designed by surgeons and anesthesiologists and utilized the Consolidated Framework For Implementation Research (CFIR) framework to implement evidence-based clinical care.[16, 17] The surgical, anesthesia, and nursing champions selected the Consolidated Framework for Implementation Research structure over other approaches as it created a format to design, evaluate, and implement evidence-based practices. In the 2 years following implementation, 35 surgeons, 164 anesthesiologists, 76 certified registered nurse anesthetists (CRNAs), and 130 anesthesia residents participated in caring for patients in the pathway.

## Material and methods

To implement ERAS Hysterectomy on March 1, 2018, the entire ERAS pathway was divided into two bundles: surgical and anesthesia. Our team utilized the Consolidated Framework For Implementation Research approach to implement each bundle and ultimately change behaviors.

To implement the surgical bundle, published data and best practice position statements were first presented in individualized educational sessions to surgeons, surgical physician assistants, and nurse practitioners. Similar individualized educational sessions were provided to surgical residents when they rotated through gynecology services. Surgical leaders publicly supported the endeavor, and a large-format grand rounds for the gynecology department was held before the pathway launched.

Designing and implementing the anesthesia bundle was more complex. For the ERAS program to be successful, all bundles would need to be consensus-driven, derived from evidence-based practices, and complement other bundles. Therefore, after reviewing the most relevant literature with the anesthesia, surgical, and nursing champions, the anesthesia bundle was created.

To add to the complexity of implementing the anesthesia bundle, outreach efforts to the 164 anesthesiologists and 76 CRNAs as well as the 130 residents who rotate through different operating theaters would be mandatory.

Since duplicating the surgical approach of hosting individualized educational sessions was neither practical nor feasible, the anesthesia bundle was introduced to this group 2 months prior to implementation via email in a communication that described the entire ERAS pathway. The message was inclusive of peer-reviewed literature and data from other hospital-specific ERAS pathways demonstrating improved patient outcomes including decreased length of stay.

Next, a large-format grand rounds presentation on ERAS Hysterectomy was given to the anesthesia department. This grand rounds occurred in the month prior to pathway implementation. Evidence supporting the new pathway was presented at this conference.

Then, on the night before a patient was scheduled to provide anesthesia for a hysterectomy, each member of the anesthesia care team (attending anesthesiologist and certified nurse anesthetist or anesthesia resident) received an email containing the slide deck that had been presented at the grand rounds and a copy of the pathway with the anesthesia bundle attached. For the next 14 months, nightly emails were sent.

Nightly emails ceased 14 months after the pathway was implemented when administrative changes within the department occurred. While the pathway was intended to be posted on a new departmental intranet, it ultimately never was. If a provider requested a copy of the pathway from anesthesia leadership, then it was emailed to that provider. However, it is important to note that after a period of intense intervention, the pathway was not easily accessible to anesthesia providers.

To further enhance compliance with the pathway, annual performance reports were emailed to anesthesia providers showing individual compliance with different elements of the pathway. The first and only report that occurred during this period was emailed to providers 15 months after the pathway was implemented. Technical constraints limited our ability to email reports more frequently. Fortunately, these constraints have been addressed and are now resolved.

**Fig. 1:**
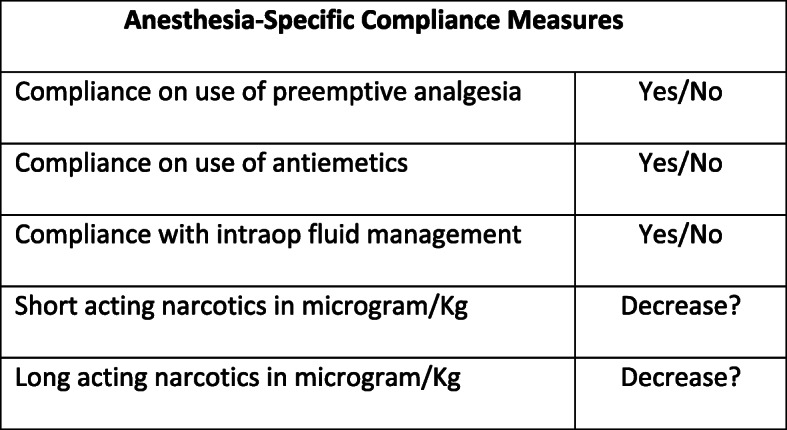
Anesthesia Compliance Measures that were included in the anesthesia bundle of our ERAS Program

To assess the impact of our approach to implement and sustain ERAS Hysterectomy, ethical approval was obtained through the Institutional Review Board (IRB) at the Massachusetts General Hospital (IRB: 2017P000443). Requirement for written informed consent was waived by the IRB. Next, 2570 consecutive charts between October of 2016 and March of 2020 were retrospectively reviewed as part of this cohort study. The objective of this study was to evaluate compliance of pre-determined ERAS metrics during intervention and post intervention periods. The outcome measures are seen in Fig. 1.

We recognize that patient comorbidities may impact patient care, and we controlled for the following demographic data: patient age, heart rate, systolic blood pressure, BMI category, and American Society of Anesthesiology (ASA) category. We also recognize that clinical factors such as hysterectomy type and subspecialty surgical division may impact our analysis. Therefore, we controlled for these factors. Finally, we controlled for compliance with four ERAS measures (when evaluating each ERAS compliance, we controlled for compliance with the other four ERAS measures) when performing our analysis. Of note, heart rate and systolic blood pressure serve as very rudimentary proxies for intra-operative pain and fluid status.

We used standard descriptive statistics to characterize the sample. Multivariable regression analyses were used to evaluate changes in ERAS measures during the intervention and post-intervention periods. For binary outcomes logistic regression was used and for continuous outcomes generalized linear models were used. A significance level of 0.05 was used to establish statistical significance and regression results are reported as odds ratio or rate ratio depending on the nature of outcome measure. All statistical analyses were performed using SAS version 9.4.[18]

## Results

A total of 1059 surgeries were performed during the baseline period, 852 surgeries during the intervention period, and 659 during the post-intervention period. See Table 1 for patient demographics.

**Table 1:** Sample Characteristics by Study Period

Sample Characteristics	Baseline (*n* = 1059)	Intervention (*n* = 852)	Post Intervention (*n* = 659)
Continuous variables, mean (std)			
Age	54.2 (13)	54.7 (13)	55.4 (13)
Heart Rate	77.9 (13.1)	77.8 (12.7)	77.9 (13.1)
SPB	125.7 (18.6)	128.7 (18.5)	128.9 (18.7)
Categorical variables, n(%)			
Hysterectomy Type			
Laparoscopic	847 (80.0)	637 (74.8)	501 (76.0)
Vaginal	86 (8.1)	67 (7.9)	47 (7.1)
Open-Debulk	72 (6.8)	78 (9.2)	49 (7.4)
Open-Plain	54 (5.1)	70 (8.2)	62 (9.4)
Section			
Endocrinology	51 (4.8)	46 (5.4)	27 (4.1)
MIGS	182 (17.2)	77 (9.0)	36 (5.5)
Oncology	541 (51.1)	438 (51.4)	378 (57.4)
Pelvic Medicine	118 (11.1)	94 (11.0)	87 (13.2)
Specialist	167 (15.8)	197 (23.1)	131 (20.0)
BMI Category			
Normal	231 (21.8)	205 (24.1)	164 (24.9)
Overweight	228 (21.5)	203 (23.8)	134 (20.3)
Obese	321 (30.3)	258 (30.3)	206 (31.3)
Unknown	279 (26.4)	186 (21.8)	155 (23.5)
ASA Category			
Healthy	91 (8.6)	61 (7.2)	30 (4.6)
Mild	706 (66.7)	521 (61.2)	362 (54.9)
Sever	193 (18.2)	159 (18.7)	165 (25.1)
Missing	69 (6.5)	111 (13.0)	102 (15.5)

Compared to the baseline period, compliance with four of five ERAS metrics measured showed statistically significant improvement during the intervention period (Table 2). Compliance with use of preemptive analgesia increased from 42 to 85% (odds ratio (OR) = 8.3, 95%CI = 6.5–10.6). Compliance with intra-op fluid management increased by 58% (OR = 1.58, 95% CI = 1.25–1.99). Dosage of short-acting narcotics decreased by 14% (rate ratio (RR) = 0.86, 95% CI = 0.82–0.90), and dosage for long-acting narcotics decreased by 9% (RR = 0.91, 95%CI = 0.82–1.00).

**Table 2:** Bivariate Comparison of Change in ERAS Measures^a^

Outcome Measure	Mean Values			*P*-Value for Differences	
	Baseline	Intervention	Post Intervention	baseline vs intervention	intervention vs postintervention
Preemptive Analgesia, n (%)	447 (42.2)	728 (85.5)	553 (83.9)	< 0.0001	0.4113
Antiemetics use, n(%)	967 (91.3)	796 (93.4)	603 (91.5)	0.0856	0.1566
Intraop fluid mngt, n(%)	325 (30.7)	392 (46.0)	281 (42.6)	< 0.0001	0.1913
Short acting narcotics (in mcg/kg), mean (std)	2.28 (1.1)	1.86 (1.0)	1.87 (1.0)	< 0.0001	0.7883
Long acting narcotics (in mcg/kg), mean (std)	10.56 (7.4)	9.03 (9.2)	8.90 (6.6)	< 0.0001	0.7532

^a^The above table provides unadjusted comparison of compliance measures between the two periods under study: baseline vs intervention and intervention vs post-intervention. Compared to the baseline period, four of the five metrics showed significant improvement during the intervention period. Moreover, all these improvements were sustained during the post intervention period (there were some marginal changes, but none of them were statistically significant). Use of antiemetics, which had already 91.3% compliance during the baseline period, didn’t show significant movement during the intervention or post-intervention period

Importantly, the improvements during the intervention period were sustained during the post-intervention period (see Table 3), as practitioners did not deviate in a statistically significant manner from practices that were established during the intervention period despite no active engagement from the administration. Compliance with use of antiemetics, which was already at 91.3% during the baseline period, did not show significant change on either the intervention or post intervention period.

**Table 3:** Multivariable Regression Results Assessing Changes in ERAS Measures During Intervention and Post Intervention Period*

ERAS Measures**	Intervention Period Relative to Baseline Period		Post Intervention Period Relative to Intervention Period	
	Estimate	95% CI	Estimate	95% CI
Preemptive Analgesia	8.29	6.49–10.59	0.84	0.63–1.12
Antiemetics Use	1.35	0.92–1.99	0.80	0.54–1.18
Intraop Fluid Management	1.58	1.25–1.99	0.80	0.64–1.01
Short Acting Narcotics	0.86	0.82–0.90	1.02	0.97–1.06
Long Acting Narcotics	0.91	0.82–1.00	0.98	0.89–1.08

*These results are based on regression models that controlled for the following covariates: age, heart rate, systolic blood pressure, BMI category (normal, overweight, obese, unknown), ASA category (healthy, mild, sever, missing), hysterectomy type (laparoscopic, vaginal, debulk-open, plain open), section (oncology, endocrinology, MIGS, pelvic medicine, specialist), ERAS Measures (in each ERAS model, we included the other remaining four. For example, when we assess use of short acting narcotics, we controlled for use of long acting narcotics, preemptive analgesia, antiemetics use and intraop fluid administration)

**The first three metrics are measured as binary (yes/no) outcomes and we used logistic regression to model them. The two last two are measured as a continuous outcome in microgram/kg and are modeled using generalized linear model with log link and gamma distribution

*** Results show, except for use of antiemetics, which was already high during the baseline period, the other four ERAS measures have significantly improved in the intervention period (relative to baseline period) and these improvements were sustained during the post-intervention period

## Discussion

Implementation of ERAS pathways can be challenging, but ultimately rewarding, as patients have fewer complications, spend less time in hospitals, and surgeries may be moved from inpatient arenas to outpatient surgical centers.[19, 20] In the US, initial approaches to optimizing perioperative care began with the perioperative surgical home.[21] These preliminary forays into collaborative, team-based approaches to surgical care led to the modern ERAS pathway.

Given that ERAS pathways, particularly in gynecology, are effective at decreasing complications, shortening hospital stays, and cutting costs, successful implementation in the current healthcare environment is of utmost importance.[22] However, pathways are only impactful if providers are compliant with them.[7] Modifying behaviors of a large group of clinicians is challenging and requires a multifaceted, sustained approach with repeated communication and follow-up.

Our team was successful because we followed a deliberate implementation framework. In the 3 months prior to the pathway launch, small-format meetings between the ERAS co-directors and the OB/GYN nursing director, the PACU staff, the post-operative floor nursing managers, and the OR nursing staff occurred. These small-format meetings created space for clinicians to become familiar with and enrolled in the new pathway.

Following the small-format meetings, but prior to the pathway launch, both the surgical and anesthesia bundles were emailed to surgeons and anesthesiologists. The entire pathway was then presented at surgical grand rounds and anesthesiology grand rounds. These large-format meetings allowed a rigorous academic discussion of the evidence behind the pathway and reinforced data that had previously been presented in both small-group discussions and email.

After the pathway was implemented, nightly emails to anesthesia providers who would care for ERAS hysterectomy patients reminded clinicians of the different elements of the pathway. This tactic continued for 14 months following implementation and further reinforced adherence to the pathway.

Finally, by providing anesthesiologists and nurse anesthetists with annual reports detailing individual compliance with different elements of the anesthesia bundle, providers were able to review their performance and compare their individual performance to their peers.

Perhaps the most controversial portion of our pathway centered on the fluid management goal of administering less than 4 mL/kg/hr. Many of the anesthesia clinicians at our institution expressed strong opinions about the quantity and timing of fluids administered, and achieving consensus on this element of the pathway was particularly difficult. However, despite the controversy surround the metric, compliance with fluid administration goals demonstrably increased over time.

There are several limitations of our study worth comment. First, as a retrospective analysis of a quality improvement project, the study is subject to selection bias and confounding bias. Our institution was in the process of developing and implementing multiple other ERAS pathways during our intervention and post-intervention periods. While we demonstrate a significantly increased compliance with our pathway using the CFIR framework, it is possible that this significance was impacted by other endeavors simultaneously occurring at the hospital.

A second limitation of our study is that the metrics related to intraoperative opiate administration were: 1) a “decrease in long-acting narcotic administration” and 2) a “decrease in short-acting narcotic administration.” This guidance allowed clinicians to use their best judgment when caring for their patients. However, it did not identify a target quantity of narcotic to administer and could have led to confusion.

A third limitation to our study is that our post-intervention period is 10 months. As anesthesia residency is 3 years, it is possible that we will not capture post-intervention activities in their entirety.

Finally, while our intention had been to ultimately post the ERAS Hysterectomy pathway on a departmental intranet, technical constraints prevented this from happening. Providers relied on their familiarity with the pathway or on previously sent emails that contained the pathway to guide their care. This oversight likely decreased compliance in the post-intervention period.

## Conclusions

ERAS pathways can be implemented and sustained in large anesthesia practices. Most of the effort to successfully implement the pathway occurs in the planning/early implementation period. Obtaining buy-in from surgeons, anesthesiologists, and nurses is key. Therefore, we recommend assembling a multidisciplinary team to examine the latest evidence before creating the pathway, and we advise hosting small format meetings with impacted stakeholders prior to launching a pathway.

Once the pathway is designed, senior leadership support is necessary. This support can occur via large-format grand rounds. After this public display of support, emailing the entire pathway and evidence supporting the pathway to all providers who will be involved in the care of this type of surgical patient is useful. This approach, when supplemented with individual emails to providers on the night before the procedures, is effective. Also, providing individual feedback on compliance with the pathway is impactful. After a certain amount of time, in our case 14 months, sending continual reminders may not be necessary. However, we do recommend posting the pathway in a central repository as a reference for providers.

As our ERAS pathways mature, we will incorporate additional reporting structures for practitioners in addition to integrating complication data into our reports. We also plan to refine various elements of the pathway as evidence-based practices improve. This will keep our providers engaged and make sure we provide the highest-quality care to our patients.

## Data Availability

The datasets used and analyzed in this study is available from the corresponding author on reasonable request.

## Abbreviations

ERAS: Enhanced Recovery After Surgery
CRNAs: Certified Nurse Anesthetists
CFIR: Consolidated Framework For Implementation Research
IRB: Institutional Review Board
ASA: American Society of Anesthesiology
